# Alternative School Breakfast Service Models and Associations with Breakfast Participation, Diet Quality, Body Mass Index, Attendance, Behavior, and Academic Performance: A Systematic Review

**DOI:** 10.3390/nu15132951

**Published:** 2023-06-29

**Authors:** Deborah A. Olarte, Marisa M. Tsai, Leah Chapman, Erin R. Hager, Juliana F. W. Cohen

**Affiliations:** 1 Center for Health Inclusion, Research and Practice (CHIRP), Merrimack College, 315 Turnpike Street, North Andover, MA 01845, USA; chapmanle@merrimack.edu (L.C.); or jcohen@hsph.harvard.edu (J.F.W.C.); 2 Division of Epidemiology, School of Public Health, University of California Berkeley, 2121 Berkeley Way, Berkeley, CA 94704, USA; marisa.tsai@berkeley.edu; 3 Department of Population, Family and Reproductive Health, Johns Hopkins Bloomberg School of Public Health, 615 N. Wolfe Street, Baltimore, MD 21205, USA; ehager1@jhmi.edu; 4 Department of Nutrition, Harvard T.H. Chan School of Public Health, Harvard University, 677 Huntington Avenue, Boston, MA 02115, USA

**Keywords:** alternative breakfast models, Breakfast After The Bell, breakfast in the classroom, second chance breakfast, grab and go meals, systematic review

## Abstract

The United States (US) School Breakfast Program provides Breakfast After The Bell (BATB) to alleviate hunger, provide nutrition, and ensure students have a healthy start to the day. This study aims to review the evidence regarding the impact of BATB on students’ diet and academic outcomes, including participation, diet quality and consumption, body mass index (BMI) and weight status, attendance, classroom behavior, and academic performance. The articles were extracted from three electronic databases and published since the start of the literature through December 2022. Studies were peer-reviewed; quantitative research articles or government reports; and conducted in public or private elementary, middle, and high schools. Quality was assessed using the Newcastle-Ottawa Scale. Thirty-seven studies were included in this review. This review found BATB increased school breakfast participation, improved diet quality, and improved classroom behavior particularly among students from racial and ethnic minority backgrounds and students eligible for free or reduced-price meals. The impact of BATB on BMI and weight status, academic achievement and attendance was mixed. This review is particularly timely given free school meals and updated school nutrition standards are being prioritized over the next decade in the US. Thus, it is important to evaluate the nutritional and educational outcomes of BATB. (PROSPERO registration: CRD42021289719).

## 1. Introduction

The United States Department of Agriculture’s (USDA) School Breakfast Program (SBP) is a nutrition assistance program that provides nearly 15 million children with a healthy breakfast [1,2]. The SBP has strict nutritional guidelines, which were updated in 2012 following the 2010 Healthy, Hunger-Free Kids Act (HHFKA) [3]. Previous studies examining school meals have demonstrated that school breakfast and lunch participation is associated with reductions in student hunger and lower food insecurity, improvements in nutritional intake, and healthier body weight for young children, particularly for low-income children, highlighting the potential role of school breakfast to improve student health [3,4,5,6].

School breakfast is traditionally served in the cafeteria before the school day commences. However, this requires students to arrive early, which is often not feasible for many students and families. In addition, many students from low-income households receiving free or reduced-price breakfast may feel stigma, which discourages them from participating in school meals [7]. Overall, this may in part explain the lower participation rates in the SBP compared with the National School Lunch Program [1]. 

A variety of efforts have been made to increase participation in the SBP, such as providing Breakfast After the Bell (BATB), which includes alternative breakfast models: Breakfast in the Classroom (BIC); Grab-and-Go (GG); and Second Chance Breakfast (SCB) [8]. These BATB initiatives aim integrate breakfast into the school day [9]. Specifically, BIC provides students breakfast at their desks during the first 15 min of class, typically while morning announcements and attendance is taken [8]. GG is a flexible model and offers students the opportunity to pick-up breakfast on their way to class, either from the cafeteria, or in coolers or carts located in the hallways [8]. SCB serves breakfast in the cafeteria or via kiosks or carts in the hallways between class periods [8]. 

To inform the adoption of BATB in schools, an understanding of the impact of these alternative breakfast models is warranted. The aim of this study was to systematically review the evidence regarding the impact of BATB on students’ nutritional and educational outcomes, including breakfast participation, diet quality and consumption, body mass index (BMI) and weight status, attendance, classroom behavior, and academic performance.

## 2. Materials and Methods

This review synthesized the evidence regarding the impact of BATB initiatives (namely BIC, GG, and SCB) on students’ outcomes related to breakfast participation, diet, and academic performance. This review was conducted in accordance with the Preferred Reporting Items for Systematic Reviews and Meta-Analyses (PRISMA) guidelines [10] and was registered with the PROSPERO International Prospective Register of Systematic Reviews (protocol registration number: CRD42021289719). 

### 2.1. Search Strategy

The articles were extracted from three electronic databases: PubMed, Education Resources Information Center (ERIC), and Scopus. The search strategy included combinations of the keywords and phrases: breakfast, school, after the bell, classroom, grab, second chance. No filters were used in the searches. All articles written in English and published since the start of the literature through December 2022 were reviewed. Included article reference lists were reviewed to identify any other applicable studies. A review of articles citing the included literature via Google Scholar was also conducted. 

### 2.2. Study Selection

Included studies were quantitative research articles that evaluated BATB interventions conducted in public elementary, middle, and high schools. Inclusion criteria were peer-reviewed studies and government reports conducted during the school year (SY). Studies that were qualitative or that examined school breakfast initiatives in pre-schools, Head Starts, colleges or weekend programs, or in locations outside the United States (US) were excluded. 

Study titles and abstracts were screened, and based on the eligibility criteria, the full texts were reviewed. Articles with unclear eligibility and discrepancies were reviewed and settled by the research team. Due to the heterogeneity of the methods and study designs used in the included studies, the data could not be combined to be analyzed as a meta-analysis. Therefore, a qualitative narrative review was used to organize and synthesize the results of the included studies. 

### 2.3. Quality Assessment

While relying on the Newcastle-Ottawa Scales (NOS) for cross-sectional and cohort studies, quality and bias were assessed [11]. The NOS scale has been adapted for different study designs, including observational and experimental studies. The studies were assessed on selection, comparability, and outcome (i.e., sample size, comparison groups, comparability of groups, measurement at baseline, objectivity of outcome measures, statistical tests used, and adjustment for confounding factors) and interpreted based on three categories: very high risk of bias (0–3 points); high risk of bias (4–6 points); and low risk of bias (7–10 points) [11]. The quality assessments of cohort and quasi-experimental studies based on the NOS are presented in Supplemental Table S1. The quality assessment of cross-sectional studies based on the NOS is presented in Supplemental Table S2. 

## 3. Results

**Figure 1:**
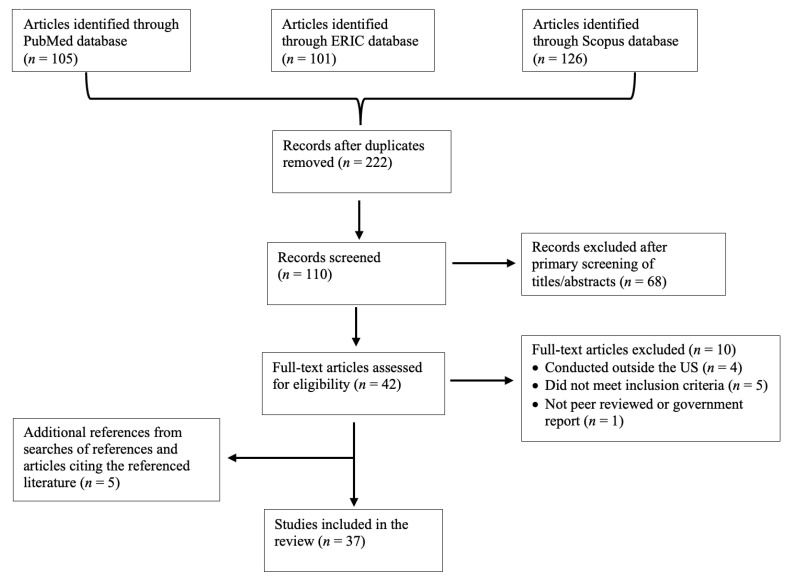
Flow chart depicting the systematic review of studies based on alternative school breakfast service models and associations with breakfast participation, diet quality, body mass index, attendance, and academic performance.

The database searches identified 332 articles, and *n*= 222 duplicate records were removed. Primary screening removed an additional 110 articles. The full text of the remaining 42 articles were examined, and ten additional full-text articles were excluded based on the inclusion criteria. An additional five articles were identified from publication references, resulting in 37 studies/government reports included in this review (Figure 1) [12,13,14,15,16,17,18,19,20,21,22,23,24,25,26,27,28,29,30,31,32,33,34,35,36,37,38,39,40,41,42,43,44,45,46,47,48]. 

Based on the NOS, the risk of bias scores ranged from 3 (very high risk of bias) [45] to 10 (low risk of bias) [11]. Approximately 76% (*n* = 28) of studies were classified as low risk of bias [12,13,14,15,16,17,18,21,23,24,26,27,28,29,30,31,32,33,34,35,37,38,41,42,43,44,47,48]. The remaining 8 studies had a high risk of bias [19,20,22,25,36,39,40,46], and one study had a very high risk of bias [45]. The studies were randomized controlled trials [16,28,30,33,34,38,41,42], quasi-experimental with and without control groups [12,13,15,18,20,21,22,23,24,25,26,29,31,32,35,36,37,39,40,43,47,48], and cross-sectional [14,17,27,44,45,46]. One study assessed trends in breakfast participation over time [19].

The included studies are presented in Table 1. The studies conducted among students in grades K-5 or K-8 were classified as elementary schools (*n* = 29), grades 6–8 grade were classified as middle school (*n* = 9), and grades 9–12 were classified as high schools (*n* = 11). Additionally, 16 studies were conducted after the implementation of the HHFKA in 2012, 16 studies were conducted prior, and 5 studies were conducted during the 2012–2013 school year when HHFKA was implemented. This was important to examine because the HHFKA implemented nutrition standards that changed the composition of school meals. 

### 3.1. Participation

One priority of providing BATB is to increase SBP participation. Among the BATB initiatives, BIC was the most studied. In total, 22 studies (including *n* = 3 government reports) assessed participation rate. By school type, there were *n* = 15 studies conducted in elementary schools, *n* = 7 in middle schools, and *n* = 9 in high schools. Eleven studies were conducted prior to the HHFKA implementation, nine studies were conducted after the implementation, and two studies were conducted during the implementation. All studies in the review found increased SBP participation. The largest increases were found among students who were eligible for free or reduced-price meals (FRPM) and in schools that implemented BIC. Five of the twenty-two studies had a high risk of bias, and one had a very high risk of bias.

Among studies that examined school meal participation, the majority were conducted in elementary schools. A 2002 cross-sectional study used a nationally representative sample of third grade students to assess SBP participation. Higher rates of participation were reported when breakfast was served in the classroom [14]. Similarly, a government report assessing a pilot program of universal free breakfast in six school districts in six states reported that BIC resulted in higher participation (65%) compared to schools that served breakfast in a non-classroom setting (28%) [35]. A separate government report built upon the same pilot program found that participation was higher for students who received BIC [18]. An additional government report, expanded on the pilot program data from the previous two reports, finding BIC increased SBP participation by 38 percentage points, or an increase in participation of 144% (SE: 2.18; *p* < 0.0001) [43]. 

In large, urban cities, where competing foods are more prevalent, BATB initiatives were also successful in improving student participation in elementary schools. One study found that during the 2012–2013 SY, the mean student participation rate was 73.7% (*p* < 0.001), whereas in schools without BIC, breakfast participation was 42.9% (*p* < 0.001) [13]. In New York City, elementary schools that offered BIC schoolwide saw an increase in participation of 33.3 percentage points (SE: 0.043; *p* < 0.0001) [21]. In Philadelphia, Pennsylvania, BIC was offered at 16 public elementary schools between 2014 and 2016. By the end of the study period, participation increased to 72% of school days in BIC schools. In comparable control schools, participation decreased to 24.9% (β = 0.33; 95% CI: 0.24–0.42) [41].

Suburban and rural areas also saw improvements in participation in elementary schools. Among fourth grade students, participation was greater with BIC compared to traditional breakfast service (*p* < 0.0001), with the largest percentage of participating students being eligible for FRPM (63.4%; *p* < 0.0001) [29]. In a cross-sectional study, BIC was positively associated with participation for elementary school students (OR: 1.49 [95% CI: 1.14–1.93] *p* < 0.01) [44]. In addition, a study conducted in three public schools in northern Nevada and a study in rural, southwest Virginia found increases in participation among elementary school children who participated in BIC. However, both had a high risk of bias [25,36].

BATB initiatives in middle school also demonstrated increases in student breakfast participation. In New York City, participation increased by 33.6 percentage points (SE: 0.032; *p* < 0.0001) in middle schools [21]. In North Carolina, GG (OR: 1.52 [95% CI: 1.01–2.28] *p* < 0.05) and SCB (OR: 2.61 [95% CI: 1.68–3.09] *p* < 0.0001) approaches were positively associated with higher odds of participation among middle school students [44]. In Pennsylvania, middle schools experienced a non-significant increase in participation, versus high schools (OR: 1.23 [95% CI: 0.88–1.71]) [48]. Another study in Pennsylvania middle schools found increases in participation, particularly in students who were not eligible for FRPM, although this study had a high risk of bias [20]. A similar study in Minneapolis, Minnesota, examined SBP participation over six weeks and found increases in student participation and increased participation among students who were eligible for FRPM. However, this study also had a high risk of bias [39].

In high school, where breakfast participation is particularly low, studies showed an increase in participation in schools where BATB initiatives were implemented. Three studies were conducted in Minnesota. A randomized control trial (RCT) in twelve high schools examining SCB found that mean participation increased from 16.3% to 25.7% (*p* = 0.004) [28]. Similarly, one study found student participation increased from 13.0% to 22.6%. Among students eligible for FRPM, participation increased from 13.9% to 30.7% (*p* < 0.001) [33]. Another study assessed school breakfast participation from 2012 to 2015, finding the median increase in the intervention group was 3% higher in GG schools by the end of the intervention period compared to 0.5% in the control group (*p* = 0.03). Over time, the intervention group increased participation to 10.3% (95% CI: 3.0–17.6) [38]. In North Carolina, BATB approaches improved participation among high school students. BIC (OR: 2.12 [95% CI: 1.20–3.75] *p* < 0.01), SCB (OR: 2.27 [95% CI: 1.66–3.09) *p* < 0.0001), and GG (OR: 1.35 [96% CI: 1.05–1.72] *p* < 0.05) were associated with a higher likelihood of participation [44]. A study conducted in a Midwest suburb found a 400% increase in school breakfast participation. However, this study had a high risk of bias [40]. Lastly, with the implementation of a morning nutrition break, a study conducted in an urban high school found 69% of students participated. This study had a very high risk of bias [45]. 

Examining BATB initiatives statewide in elementary, middle, and high schools showed that they were also successful at improving student participation. A study conducted in Missouri from 2016 to 2020 found participation increased by 1.4 percentage points, equating to approximately 756,000 more breakfasts served per year (*p* < 0.05). In addition, in schools serving BATB, there was a 10-percentage-point increase in participation among FRPM-eligible students [26]. In North Carolina, a similar study examined district-level participation over time between the 2007–2008 and 2014–2015 SY, and in districts that adopted BIC, there was a 1.1-percentage-point increase (SE: 0.4; *p* = 0.002) equivalent to over 16,000 more students receiving breakfast [19]. In Pennsylvania, a study with 194 schools between the 2017–2018 and 2018–2019 SY found greater participation in elementary schools that implemented BIC compared to high schools. Specifically, elementary students that received BIC had an increased likelihood of participating in breakfast than those who did not (OR: 1.96 [95% CI: 1.19–3.23] *p* < 0.0001) [48].

### 3.2. Consumption and Diet Quality 

Breakfast participation is the first step, but to improve diet quality, students need to eat the breakfast as well. Nine studies assessed the impact of BATB on breakfast consumption and dietary quality, three of which were government reports. Specifically, one study assessed consumption, and eight studies assessed diet quality. All nine studies were conducted with elementary school students and assessed BIC. Seven studies relied on data prior to the implementation of the HHFKA. Three studies had a high risk of bias.

Prior to the HHFKA, studies suggest students are more likely to have increased caloric consumption, likely from eating breakfast at home and at school [17,18,42,46]. However, one study found students who ate BIC had the highest Healthy Eating Index-2010 score compared to students who ate breakfast in the cafeteria or SCB and were more likely to have increased total and whole fruit consumption and decreased consumption of empty calories [42]. Similarly, a government report found BIC students were more likely to eat a substantive breakfast and consume more whole grains compared to students who ate in the cafeteria [18]. In Maryland, there were fewer teacher reports of student hunger when BIC was implemented (48% and 60% in continuing and new BIC schools, respectively, versus 68% and 77% in drop-out or control schools, respectively (*p* < 0.001)) [37]. One study found little to no difference in caloric consumption after assessing nutritional intake over three two-week periods among elementary students in Nevada. This study had a high risk of bias [36]. 

Similar results were seen among studies conducted post-HHFKA implementation. Students who ate BIC consumed a nutritionally substantive breakfast and had better dietary outcomes than students who ate breakfast in the cafeteria or from home [16,43]. Only one study examined consumption through food waste in elementary schools implementing BIC. Food waste was assessed pre- and postimplementation of BIC using the quarter-waste method (marks food wasted as either 0%-none remaining, 25%, 50%, 75%, or 100%-all remaining). With BIC, food waste decreased from 43% to 38.5% (*p* < 0.01). Entrée waste decreased the most from 44.8% to 21.4% (*p* = 0.001). However, this study had a high risk of bias [25]. 

### 3.3. BMI and Weight Status

There is growing concern over students eating breakfast at home and at school, and thus increasing energy intake leading to increased weight and BMI. In total, four studies were conducted examining the impact of BATB on student BMI and weight status. One study was conducted in both elementary and middle schools. Two of the four studies were conducted prior to the HHFKA implementation. Across studies, there was mixed evidence on the impact of BATB on student BMI and weight status. Two studies found increases in student BMI and weight status, while two found no evidence of an increase in weight. All studies had a low risk of bias.

Prior to the HHFKA, a study in both elementary and middle schools found no evidence of an increase on student BMI after BIC implementation in both types of schools [21]. Conversely, a repeated cross-sectional study examined the relationship of raw BMI and breakfast location over three SYs and found that BIC was significantly associated with higher BMI (*p* = 0.012) among fourth grade students from one school district. However, BIC was not significantly associated with the BMI categories (i.e., healthy weight, overweight, obese, and severely obese (OR: 1.31; *p* = 0.054)) [17]. 

Following HHKFA implementation and using data from the Early Childhood Longitudinal Studies-Kindergarten (ECLS-K), one study found an increase in the prevalence of overweight but not obesity among students in four states over time. The risk of overweight increased specifically for females. Additionally, point estimates were higher among children from racial and ethnic minority backgrounds and low-income backgrounds [12]. In an urban, low-income community, the effects of BIC on overweight and obesity were examined over 2.5 years. There was no statistically significant difference in the combined incidence of overweight and obesity after BIC implementation. However, in intervention versus control schools, the data suggested increases in the incidence of obesity (11.6% vs. 4.4% [95% CI 1.87–5.73]) and prevalence of obesity (28.0% vs. 21.2% [95% CI 1.08–1.89]) after BIC implementation [41]. 

### 3.4. Attendance

SBP participation may increase student attendance, particularly among older students. In total, ten studies examined attendance with BATB; three were conducted in middle schools and one in a high school. Four studies were conducted prior to HHFKA implementation, four after implementation, and two during. Overall, the results were mixed. Of the ten studies conducted in elementary schools, six found increased student attendance after the implementation of BATB, while four found no association. The studies including middle and high schools found increases in student attendance. All ten studies had a low risk of bias. 

Half of elementary school studies found improvements in attendance. One government report conducted in Maryland found improvements in attendance with BIC [37]. Another government report found a 1.05-percentage-point increase in attendance after three years, but pooled impacts across all three years were small and not statistically significant [43]. In a large, urban school district, one study found a small but statistically significant increase in attendance (i.e., 95.5% with BIC versus 95.3%, with breakfast served in the cafeteria (*p* = 0.004)) [13]. Additionally, using a nationally representative sample during the 2010–2011 SY, another study found students had fewer absences (−0.50 [SE: 0.24] *p* < 0.05) and a lower likelihood of chronic absenteeism (−0.04 (SE: 0.02) *p* < 0.05) with BIC [27]. However, the remaining four studies conducted in elementary schools showed that serving BIC had no impact on absenteeism [15,21,31,34]. 

Studies that examined attendance across elementary, middle, and high school also saw improvements in attendance. Using data from elementary and middle schools in a low-income urban area in the northeast US from 2013 to 2015, found BIC to decrease a total number of absences per SY (3.08 days in 2015 to 2.89 days in 2016; *p* = 0.034) [47]. Similarly, one study found serving BATB lead to an improvement in student attendance and chronic absenteeism decreasing by three percentage points (*p* < 0.01). The greatest effect was seen among high school students where chronic absenteeism decreased by seven percentage points (*p* < 0.001) [32]. 

### 3.5. Behavior

Food insecurity and hunger could impact students’ emotional and mental well-being and cause poor behavior in the classroom. BATB initiatives may be helpful in decreasing poor student behavior. Four studies examined the effect of BIC initiatives on student behavior in elementary and middle school. Two studies were conducted prior to HHFKA implementation, one study after, and one during implementation. Overall, results indicate improvements in student behavior, particularly among racial and ethnic minority students. All studies had a low risk of bias. 

A government report conducted in Maryland found improved student attitudes, behavior and attentiveness with BIC [37]. A study using data from the Arkansas Department of Education found BATB reduced poor student behavior, including aggressive behavior and misconduct in school. Specifically, there were on average 0.249 fewer behavior violations per grade (*p* < 0.05) and students who came from racial and ethnic minority and/or low-income backgrounds saw greater improvements in behavior [24]. Similarly, a study examined the effects of BIC versus a traditional school breakfast program over two SYs in nine elementary and middle schools in low-income urban areas. While the rates of suspensions increased in both groups, the rate of increase was lower in the BIC group compared with the traditional cafeteria [47]. In contrast, a government report found no associations overall, although poor student behavior decreased among children who were racial and ethnic minorities (−0.18 [SE: 0.07] *p* < 0.0001) [43]. 

### 3.6. Academic Achievement

Students who begin school days with a substantive breakfast may be better focused, thus improving their academic achievement. Twelve studies examined academic achievement; eleven studies were conducted with elementary-school-aged students (one with elementary and middle schools,) and one was conducted in a high school. Four studies were conducted prior to HHFKA implementation, and three were conducted during implementation. The elementary and middle school studies examined student achievement through reading and math standardized test scores. The high school study examined student grade point average. Overall, the results were mixed. One study had a high risk of bias. 

Four studies found a decrease in math scores. A comparison of traditional school breakfast to BIC found student-level math scores were on average 0.05 SD lower for boys who would likely participate in BIC (*p* = 0.045) [15]. Two studies assessed academic achievement using data from 2015 to 2019. One found a reduction of 0.02 SD in standardized math test scores at BATB schools (95% CI −0.042, −0.001) [22]. The second study conducted between 2009 and 2019 also found reductions in math scores with BATB, particularly in schools with <600 students. However, this study had a high risk of bias, and the effects were not statistically significant by the time the first fully exposed cohort of kindergarteners reached third grade [23]. Additionally, in comparison to schools where breakfast was served in the cafeteria, in schools receiving BIC, math scores decreased slightly after 1.5 years (−0.14 ± 0.05, *p* = 0.004) and 2.5 years (−0.20 ± 0.07, *p* = 0.005) [34]. Five studies found no statistically significant differences in standardized test scores [13,30,35,37,43]. However, one study found a 0.007 SD increase in math scores (*p* < 0.05) among middle school students [21].

Two studies conducted in elementary schools found improvements in academic achievement. One study found reading, math and science test scores improved with BATB between 2011 and 2016. While the overall results were not statistically significant, science scores increased by 13.3% (*p* < 0.05) [12]. Improvements were also seen in reading and science scores among Black and Hispanic students [12]. In another study, math scores increased by 0.09 SD, and reading scores increased by 0.06 SD (*p* < 0.001) from 2004 to 2010. In addition, test scores increased by 0.12 standard deviations (*p* < 0.0001) in math and 0.09 standard deviations (*p* < 0.05) in reading for Hispanic students [31]. For students eligible for free school meals, math scores increased 0.13 standard deviations (*p* < 0.0001) [31]. 

## 4. Discussion

This study systematically reviewed the literature on BATB approaches on student outcomes: participation, diet and consumption, BMI and weight status, attendance, student behavior, and academic achievement. The most common BATB approach was BIC. This review found improvements in school breakfast participation, diet quality, and student behavior with BATB approaches. Research examining the impact of BIC on BMI and weight status, academic achievement and attendance was mixed. Nevertheless, the evidence shows positive associations between offering BATB and a variety of student nutrition and behavioral outcomes, particularly among underserved populations.

This review is particularly timely given the current policy context in the US. During the 2022–2023 SY, Universal Free School Meals (UFSM), which provided school breakfast and lunch to all students during the COVID-19 pandemic was deimplemented, and the previous FRPM policy was reinstated. Many schools also reverted to a traditional breakfast service model in the cafeteria. Therefore, many students who previously had access to school breakfast may no longer receive a healthy meal to start the school day. However, the Biden administration is prioritizing UFSM over the next ten years [49]. The USDA has also proposed a reduction of added sugars in school meals commencing in the fall of 2025 which will impact the quality of breakfast served through the SBP [50,51]. Thus, it is important to evaluate the outcomes of BATB as these policy changes eventuate. 

This study has several strengths and limitations. Due to the heterogeneity and inconsistencies of the methods and study designs used among the included studies, the data could not be combined to be reanalyzed as a meta-analysis. Additionally, the search terms used for this systematic review may not have retrieved all articles relevant to BATB in the US. Therefore, this study’s conclusions are limited to the publications retrieved during the search process. This review only included studies conducted in the US. While the results may be generalizable to other developed countries with alternative breakfast-serving models in schools, future studies should examine these programs in other countries. Most studies had a low risk of bias. However, some studies had a high risk of bias, and one study had a very high risk of bias. Extracting the studies with high risk of bias, and the study with very high risk of bias, did not change the conclusions. Additionally, eight studies were randomized controlled trials, and numerous studies were quasi-experimental, relying on the pre/postdesign with comparison groups or natural experiments. However, due to their real-world settings, natural experiments often have better generalizability. Most of the studies were conducted prior to the HHFKA. More research is needed to understand the impact of BATB in the context of the HHFKA, particularly on BMI and weight status as well as academic achievement. Additionally, most of the studies included in this review were conducted in elementary schools. More research is needed within middle and high schools.

## 5. Conclusions

The evidence suggests BATB approaches have the potential to increase school breakfast participation, which can lead to improvements in students’ nutrition through consumption and diet quality, and improvements in students’ classroom behavior. There was mixed evidence for their impact on BMI and weight status, attendance, and academic achievement. Additional research is needed in these areas. Investing in a school meals program that makes school breakfast easily accessible for all students and provides valuable nutrition to students will have a beneficial impact on student health and education.

## Figures and Tables

**Table 1:** Characteristics and details of the studies included in the systematic review of studies based on alternative school breakfast service models and associations with breakfast participation, diet quality, body mass index, attendance, and academic performance.

	Author, Year	School Type; Location	Breakfast Exposure	Outcome	Results	Bias Score	Pre/Post HHFKA
1	Abouk, 2022 [12]	Elementary School; Colorado, Nevada, New Mexico, West Virginia, Dallas, South Dakota	BATB	BMI/Weight (−) Achievement (+)	BMI/Weight: There was a 3.36 percentage point increase in prevalence of overweight and obesity. There was an 11.6% increase in the prevalence of children who are overweight after BATB is implemented. There was no increase in obesity prevalence. The effect was seen mostly in girls, low-income children, and Black children. Achievement: test scores are positive but not statistically significant	Low	Post
2	Anzman-Frasca, 2015 [13]	Elementary School; Large urban school district	BIC	Participation (+) Attendance (+) Achievement (+/−)	Participation: The BIC program increased breakfast participation (mean participation rate of 73.7% in BIC group vs. 42.9% in non-BIC group). Attendance: There were statistically significantly greater attendance rates (95.5% in BIC group vs. 95.3% in non-BIC group; *p* = 0.004). Achievement: There were no differences in standardized test performance in math or reading.	Low	Post
3	Bartfeld, 2010 [14]	Elementary School; Nationally representative sample	BATB	Participation (+)	Participation: Participation is associated with where breakfast is offered (classroom vs. cafeteria).	Low	Pre
4	Bartfeld, 2019 [15]	Elementary School; Wisconsin	BATB	Attendance (−) Achievement (+/−)	Attendance: No significant associations of BIC with attendance. Achievement: BIC was associated with lower math scores (0.05 SD; *p =* 0.045) among boys. BIC was not associated with academic achievement in any other models.	Low	Pre/post
5	Bauer, 2020 [16]	Elementary School; Philadelphia, Pennsylvania	BIC	Diet quality (+)	Diet quality: More students in intervention schools consumed 100% juice. Fewer intervention students consumed SSBs and foods high in saturated fats and added sugar. A greater percentage of intervention students consumed breakfast at school (44.3% vs. 13.1% in control). BIC did not reduce breakfast skipping in intervention and control (22.9% intervention; 24.2% control) and increased the number of locations students ate (29.1% in intervention; 21.2% in control).	Low	Post
6	Baxter, 2010 [17]	Elementary School; South Carolina	BIC	Participation (+) BMI/Weight (−)	Participation: BIC increased participation (30.2 percentage point increase where BIC is offered schoolwide). BMI/Weight: Breakfast location was significantly associated with BMI (BMI was larger for BIC than cafeteria).	Low	Pre
7	Bernstein, 2004 [18]	Elementary School; Columbiana, AL;Phoenix, AZ; Santa Rosa, CA; Boise, ID; Wichita, KS; Gulfport, MS	BIC	Participation (+) Diet quality (+)	Participation: Participation was higher for students with BIC. Diet quality: Students with BIC were more likely to eat a substantive breakfast than students who ate in the cafeteria; these students had higher intakes of energy, total fat, sodium but less of certain vitamins and minerals and ate more grain products.	Low	Pre
8	Bullock, 2021 [19]	E/M/H; North Carolina	BIC	Participation (+)	Participation: There was a statistically significant increase (1.1 percentage points) after BIC (equivalent to 16,243 more students participated in the SBP in NC).	High	Pre/post
9	Conklin, 2004 [20]	Middle School; Pennsylvania	GG	Participation (+)	Participation: There was an increase in participation in all three tiers of students (free, reduced and paid) with largest participation seen among the paid students (t (24d.f.) = 22.96; *p* < 0.000).	High	Pre
10	Corcoran, 2016 [21]	E/M; New York, New York	BIC	Participation (+) BMI/Weight (+/−) Attendance (+/−) Achievement (+/−)	Participation: BIC increased participation (30.2 percentage point increase where BIC is offered schoolwide). BMI/Weight: There was no evidence of increases in BMI (point estimates suggest lower BMI in schools offering BIC but effects are small and statistically imprecise). Attendance: No evidence of improvements in attendance. Achievement: No evidence of improvements in academic achievement.	Low	Pre/post
11	Cuadros-Menaca, 2022 [22]	Elementary School; Arkansas	BATB	Achievement (+/−)	Achievement: There was limited evidence that BATB impacts academic achievement. BATB reduces math scores by 0.02 SD with no evidence that BATB impacts ELA scores.	Low	Post
12	Cuadros-Menaca, 2022 [23]	Elementary School; Arkansas	BATB	Behavior (+)	Behavior: There was no significant difference in likelihood of poor student behavior. However, BATB reduces the number of all types of poor student behavior (0.249 fewer infractions per grade). When focusing on specific behaviors, BATB schools meaningfully reduces the incidences of extensive and intensive misconduct. No impact on aggressive behaviors.	Low	Pre/post
13	Cuadros-Menaca, 2022 [24]	Elementary School; Arkansas	BATB	Achievement (+/−)	Achievement: There was little evidence of positive or negative effects on academic achievement. Certain schools (i.e., GNG and schools with <600 students showed statistically negative effects on math after BATB adoption. Effects were no longer present by the time the first fully exposed BATB cohort reached third grade.	High	Pre/post
14	Farris, 2019 [25]	Elementary School; Southwest Virginia	BIC	Participation (+) Diet quality (+)	Participation: Student participation increased (meals served pre-BIC: 861; meals served post-BIC: 952; significance not calculated). Diet quality: Food waste decreased 43–38.5% (*p* < 0.01). Entrée waste decreased the most (44.8–21.4%).	High	Post
15	Ferris, 2022 [26]	E/M/H; Missouri	BATB	Participation (+)	Participation: BATB adoption increased (36.6% in 2017/18 to 46.3% in 2019/20). FRP breakfast participation increased in schools with BATB approaches. BATB adoption increased FRP breakfasts served by 1.4 percentage points (*p* < 0.05).	Low	Post
16	Gottfried, 2022 [27]	Elementary School; Nationally representative sample	BIC	Attendance (+)	Attendance: Students had fewer absences and a decrease in chronic absenteeism in schools where BIC was implemented.	Low	Pre
17	Grannon, 2019 [28]	High School; Minnesota	SCB/GG	Participation (+)	Participation: Post-SCB participation in SBP increased from an average of 16.3% to 25.7% (*p* = 0.004) where both before and second chance were combined, but no significant change in schools where breakfast was served only before school. Students who took the bus to school were more likely to participate in SCB. Students who took a car, bike or walked to school were 4.5% less likely to participate (*p* = 0.006).	Low	Post
18	Guinn, 2014 [29]	Elementary School; South Carolina	BIC	Participation (+)	Participation: Free-meal status had the largest participation (63.4%). Overall, 71% of students participated in BIC, whereas 38% participated in the cafeteria.	Low	Pre
19	Hearst, 2019 [30]	High School; Minnesota	SCB/GG	Achievement (+/−)	Achievement: There was no statistically significant change in GPA between the intervention and control groups. At 1-year follow-up there was a statistically significant increase in GPA among low-income students.	Low	Post
20	Imberman, 2014 [31]	Elementary School; Large urban district, Southwest, US	BIC	Attendance (−) Achievement (+)	Attendance: No evidence of impact on attendance. Achievement: BIC raised math and reading achievement by 0.09 and 0.06 SD, respectively. Greatest effect seen in low-performing, FRM-eligible, Hispanic and low-BMI students. BIC did not influence standardized reading exam scores after 2.5 years. Students in BIC schools had lower math exam scores after 2.5 years in comparison to students in control schools.	Low	Pre
21	Kirksey, 2021 [32]	E/M/H; Colorado, Nevada	BATB	Attendance (+)	Attendance: Schools serving BATB experienced declined in chronic absenteeism. Most strongly seen in high schools, schools with high rates of breakfast participation, schools serving UFM and suburban schools.	Low	Post
22	Larson, 2018 [33]	High School; Minnesota (rural)	GG	Participation (+)	Participation: SBP participation increased from 13% to 22.6%. Student-level increases in SBP participation increased from 7.6% to 21.9%. Increases were also seen in students who were eligible for FRPM (13.9% to 30.7%) and among those who were not eligible (4.3% to 17.2%).	Low	Post
23	Luan, 2021 [34]	Elementary School; Philadelphia, Pennsylvania	BIC	Attendance (−) Achievement (+/−)	Attendance: BIC did not influence attendance. Achievement: BIC did not influence standardized reading exam scores after 2.5 years. Students in BIC schools had lower math exam scores after 2.5 years in comparison to students in control schools.	Low	Post
24	McLaughlin, 2002 [35]	Elementary School; Columbiana, AL, Phoenix, AZ, Santa Rosa, CA, Boise, ID, Wichita, KS, Gulfport, MS	BIC	Participation (+) Achievement (+/−)	Participation: BIC resulted in higher participation (~65%) compared to schools with breakfast in other locations. Achievement: No difference in reading and math scores.	Low	Pre
25	Moeltner, 2018 [36]	Elementary School; Reno/Sparks, Nevada	BIC	Participation (+) Diet quality (+/−)	Participation: BIC adds an additional 35–45% increase in average daily participation for a close to 100% participation rate in three schools. Diet quality: There was no difference in caloric consumption compared to baseline.	High	Pre
26	Murphy, 2000 [37]	Elementary School; Maryland	BIC	Diet quality (+) Attendance (+) Behavior (+) Achievement (+)	Diet quality: BIC was associated with improvements in student complaints of hunger. Attendance: BIC was associated with decreased absenteeism. Behavior: BIC was associated with improve student behavior. Achievement: BIC was associated with improved student achievement.	Low	Pre
27	Nanney, 2011 [38]	Middle School; Minneapolis, Minnesota	BIC/GG	Participation (+)	Participation: Significant increase in intervention group. SBP participation from 0.74 days per week to 1.21 days per week (*p* < 0.0001).	High	Pre
28	Nanney, 2019 [39]	High School; Minnesota	GG	Participation (+)	Participation: There was a 3% increase in participation in intervention group with increase to 10.3% at follow-up.	Low	Post
29	Olsta, 2013 [40]	High School; Midwestern suburb	SCB/GG	Participation (+)	Participation: School breakfast participation increased 400% (i.e., 80 students participated the first week of school; after implementation, participation increased to 368 students between January and March 2012).	High	Pre
30	Polonsky, 2019 [41]	Elementary School; Philadelphia, Pennsylvania	BIC	Participation (+) BMI/Weight (+/−)	Participation: SBP participation in intervention (BIC) schools was 53.8% compared with 24.9% in control schools. BMI/Weight: There was no difference in combined incidence of overweight and obesity (11.7% vs. 9.1%). Comparing intervention and control schools, respectively, incidence (11.6% vs. 4.4%) and prevalence (28.0% vs. 21.2%) of obesity were higher in intervention schools after 2.5 years.	Low	Post
31	Ritchie, 2015 [42]	Elementary School; San Diego and Imperial Counties, California	BIC	Diet quality (+)	Diet quality: BIC was associated with fewer students not eating breakfast but more students eating breakfast at home and school. BIC did not have a higher energy intake or higher daily energy intake. BIC had higher diet quality.	Low	Pre
32	Schanzenbach, 2014 [43]	Elementary School; Columbiana, AL, Phoenix, AZ, Santa Rosa, CA, Boise, ID, Wichita, KS, Gulfport, MS	BIC	Participation (+) Diet quality (+) Attendance (+) Behavior (+) Achievement (+/−)	Participation: UFB and BIC increased participation. BIC increased participation by 38 percentage points or 144% increase in participation. Diet quality: Little evidence of improvement in nutrition. BIC increases likelihood of breakfast consumption. Attendance: Attendance rates increased 1.05 percentage points in year 3. However, all three years combined, there was no significant difference. Behavior: Little evidence of improvement in student behavior. Achievement: Little evidence of improvement in achievement.	Low	Pre
33	Soldavini, 2019 [44]	E/M/H; North Carolina	BIC	Participation (+)	Participation: BIC and GNG were positively associated with SBP participation for elementary and high school students. GNG was positively associated with SBP participation for middle and high school students. UFM was positively associated with SBP participation alone and in combination with BIC, second chance and BIC + GNG.	Low	Post
34	Sweeney, 2006 [45]	High School; Urban	SCB	Participation (+)	Participation: 69% of students reported participating at least one day per week.	Very high	Pre
35	Van Wye, 2013 [46]	Elementary School; New York, New York	BIC	Diet quality (+)	Diet quality: Students were more likely to report eating in 2 or more locations during the morning; BIC students ate an estimated 95 more kcal per morning than students not offered BIC.	High	Pre
36	Walker, 2021 [47]	E/M; Northeastern city	BIC	Attendance (+) Behavior (+)	Attendance: Absences were lower for students receiving BIC. Behavior: School suspensions were lower.	Low	Post
37	Yeh, 2022 [48]	E/M/H; Pennsylvania	BATB	Participation (+)	Participation: Breakfast participation in schools that initiated BATB increased. Largest increases were in schools that implemented BIC.	Low	Post

## Data Availability

No new data were created or analyzed in this study. Data sharing is not applicable to this article.

## Supplementary Materials

The following supporting information can be downloaded at: https://www.mdpi.com/article/10.3390/nu15132951/s1, Table S1: Quality assessment of cohort and quasi-experimental studies based on the Newcastle-Ottawa Scale for studies based on alternative school breakfast service models and associations with breakfast participation, diet quality, body mass index, attendance, and academic performance; Table S2: Quality assessment of cross-sectional studies based on the Newcastle-Ottawa Scale for studies based on alternative school breakfast service models and associations with breakfast participation, diet quality and attendance.
