# Supplementary material for: Alternative School Breakfast Service Models and Associations with Breakfast Participation, Diet Quality, Body Mass Index, Attendance, Behavior, and Academic Performance: A Systematic Review
Source: Nutrients. 2023 Jun 29;15(13):2951. doi: 10.3390/nu15132951 (PMC10346933; doi:10.3390/nu15132951)
Supplement: Supplementary file 1 [file nutrients-15-02951-s001.zip › Table S1.pdf]

Table S1. Quality assessment of cohort and quasi-experimental studies based on the Newcastle-Ottawa Scale for studies based on alternative school breakfast service models.

Author, Year	Satisfactory Sample[1] (++)	Comparison Group[2] (+)	Comparability of subjects; confounding factors controlled[3] (++)	Outcome measured at baseline[4] (+)	Assessment of outcome[5] (++)	Statistical test[6] (++)	Total Score (max 10)	Risk of bias[7]
Abouk, 2022 [12]	++	+	++	+	++	++	10	Low
Anzman-Frasca, 2015 [13]	++	+	++	+	++	++	10	Low
Bartfeld, 2019 [15]	++	+	++	+	++	++	10	Low
Bauer, 2020 [16]	++	+	++	+		++	8	Low
Bernstein, 2004 [18]	++	+	+	+	+	+	7	Low
Bullock, 2021 [19]	++	+	++	+	++	+	9	Low
Conklin, 2004 [20]	+			+	++	+	5	High
Corcoran, 2016 [21]	++	+	++	+	++	++	10	Low
Cuadros-Menaca, 2022 [22]	++	+	++	+	++	++	10	Low
Cuadros-Menaca, 2022 [23]	++	+	+	+	++	++	9	Low
Cuadros-Menaca, 2022 [24]	++	+	+	+	+		6	High
Farris, 2019 [25]	++			+	+	+	5	High
Ferris, 2022 [26]	++	+	++	+	++	++	10	Low
Grannon, 2019 [28]	++	+	+	+	++	++	9	Low
Guinn, 2014 [29]	++	+	+		++	++	8	Low
Hearst, 2019 [30]	++	+	++	+	++	++	10	Low
Imberman, 2014 [31]	++	+	++	+	++	++	10	Low
Kirksey, 2021 [32]	++	+	++	+	++	++	10	Low
Larson, 2018 [33]	++	+	+	+	++	++	9	Low
Luan, 2021 [34]	++	+	++	+	++	++	10	Low
McLaughlin, 2002 [35]	+	+	++	+	++	++	9	Low
Moeltner, 2018 [36]	+	+	+	+	+	+	6	High
Murphy, 2000 [37]	++	+		+	++	+	7	Low
Nanney, 2011 [39]	+			+	++	++	6	High
Nanney, 2019 [38]	++	+	++	+	++	++	10	Low
Olsta, 2013 [40]	+			+	++		4	High
Polonsky, 2019 [41]	++	+	++	+	++	++	10	Low

Ritchie, 2015 [42]	++	+	++	+	+	++	9	Low
Schanzenbach,2014 [43]	++	+	++	+	++	++	10	Low
Walker, 2021 [47]	++	+	++	+	++	+	9	Low
Yeh, 2022 [48]	++	+	++	+	++	+	9	Low

[1] **Sample size satisfactory:** Satisfactory simple size (>100 units of analysis (e.g., trays, students, classrooms, schools) AND three or more schools in the intervention condition (++), Satisfactory simple size (>100 units of analysis (e.g., trays, students, classrooms, schools) OR three or more schools in the intervention condition (+), versus no information provided or not satisfactory (<100 participants and fewer than three schools in the intervention condition).

[2] **Comparison group**: An unexposed group serves as a comparison for the intervention condition (+) versus no comparison group.

[3] **Comparability of groups; Confounding factors controlled:** Comparability of subjects in different outcome groups and analyses adjusted for relevant predictors/risk factors/confounders, including repeated measures, where appropriate (++), adjusted for some but not all relevant predictors/risk factors/ confounders (+), versus information not provided or analyses not adjusted for relevant predictors/risk factors/ confounders.

[4] **Outcome measured at baseline:** Baseline measurements collected (+) versus no baseline assessments.

[5] **Assessment of outcome:** Objective assessment (plate waste) (++), validated non-objective measure (visual estimation; dietary recall) (+), versus non-objective and non-validated measure (aggregate plate waste is not a valid approach).

[6] **Statistical test:** Statistical tests used to analyze the data clearly described and appropriate, measures of association presented include confidence intervals and/or probability level (p value) AND statistical tests account for clustering of observations, where appropriate (++), Statistical tests used to analyze the data clearly described and appropriate, measures of association presented include confidence intervals and/or probability level (p value), OR statistical tests account for clustering of observations, where appropriate (+), versus statistical tests not appropriate, not described, or incomplete.

[7] **Total score for the Newcastle–Ottawa Scale (NOS) is attributed to a following categories:** very high risk of bias (0–3 NOS points), high risk of bias (4–6 NOS points), and low risk of bias (7–10 NOS points)
