# Supplementary material for: Alternative School Breakfast Service Models and Associations with Breakfast Participation, Diet Quality, Body Mass Index, Attendance, Behavior, and Academic Performance: A Systematic Review
Source: Nutrients. 2023 Jun 29;15(13):2951. doi: 10.3390/nu15132951 (PMC10346933; doi:10.3390/nu15132951)
Supplement: Supplementary file 1 [file nutrients-15-02951-s001.zip › Table S2.pdf]

Table S2. Quality assessment of cross-sectional studies based on the Newcastle-Ottawa Scale for studies based on alternative school breakfast service models.

**Author, Year**	**Representative Sample[1] (+)**	**Sample Size Justified[2] (+)**	**Ascertainment of exposure/risk factor[3] (++)**	**Non-respondents[4] (+)**	**Comparability of subjects; confounding factors controlled[5] (++)**	**Assessment of outcome[6] (++)**	**Statistical test[7] (+)**	**Total Score (max 10)**	**Risk of bias[8]**
Bartfeld, 2010 [14]	+	+	++		++		+	7	Low
Baxter, 2010 [17]	+	+	++		++	++	+	9	Low
Gottfried, 2022 [27]	+	+	++		++	++	+	9	Low
Soldavini, 2019 [44]	+	+	++		+	++	+	8	Low
Sweeney, 2006 [45]	+	+		+				3	Very high
Van Wye, 2013 [46]	+	+	+	+	+	+		6	High

[1] **Representative sample:** Evidence the sample is representative of target population (+) versus convenience sample or no description.

[2] **Sample size justified:** Justification provided or satisfactory sample size (>100 participants) (+) versus no information provided or not satisfactory (<100 participants).

[3] **Ascertainment of exposure/risk factor:** Objective assessment (++), validated non-objective measure (+), versus non-objective and non-validated measure.

[4] **Non-respondents:** Proportion of target population recruited attained pre-specified target or basic summary of non-respondent characteristics in sampling frame provided (+) versus unsatisfactory recruitment rate or no summary data on non-respondents or no information provided.

[5] **Comparability of subjects in different outcome groups; Confounding factors controlled:** Comparability of subjects in different outcome groups on the basis of design/analyses or analyses adjusted for relevant predictors/risk factors/confounders (++) versus information not provided or analyses not adjusted for all relevant confounders/risk factors.

[6] **Assessment of outcome:** Objective assessment (++), validated non-objective measure (+), versus non-objective and non-validated measure.

[7] **Statistical test:** Statistical tests used to analyse the data clearly described and appropriate and measures of association presented include confidence intervals and/or probability level (p value) (+) versus statistical tests not appropriate, not described, or incomplete

[8] **Total score for the Newcastle–Ottawa Scale (NOS) for assessing the quality of non-randomized studies is attributed to a following categories:** very high risk of bias (0–3 NOS points and/or no statistical analyses conducted), high risk of bias (4–6 NOS points), and low risk of bias (7–10 NOS points)
